# Chemokine Mediated Monocyte Trafficking into the Retina: Role of Inflammation in Alteration of the Blood-Retinal Barrier in Diabetic Retinopathy

**DOI:** 10.1371/journal.pone.0108508

**Published:** 2014-10-20

**Authors:** Sampathkumar Rangasamy, Paul G. McGuire, Carolina Franco Nitta, Finny Monickaraj, Sreenivasa R. Oruganti, Arup Das

**Affiliations:** 1 Department of Cell Biology & Physiology, University of New Mexico School of Medicine, Albuquerque, New Mexico, United States of America; 2 Department of Surgery, University of New Mexico School of Medicine, Albuquerque, New Mexico, United States of America; 3 New Mexico VA Health Care System, Albuquerque, New Mexico, United States of America; 4 Department of Molecular Genetics and Microbiology, University of New Mexico School of Medicine, Albuquerque, New Mexico, United States of America; Cedars-Sinai Medical Center; UCLA School of Medicine, United States of America

## Abstract

Inflammation in the diabetic retina is mediated by leukocyte adhesion to the retinal vasculature and alteration of the blood-retinal barrier (BRB). We investigated the role of chemokines in the alteration of the BRB in diabetes. Animals were made diabetic by streptozotocin injection and analyzed for gene expression and monocyte/macrophage infiltration. The expression of CCL2 (chemokine ligand 2) was significantly up-regulated in the retinas of rats with 4 and 8 weeks of diabetes and also in human retinal endothelial cells treated with high glucose and glucose flux. Additionally, diabetes or intraocular injection of recombinant CCL2 resulted in increased expression of the macrophage marker, F4/80. Cell culture impedance sensing studies showed that purified CCL2 was unable to alter the integrity of the human retinal endothelial cell barrier, whereas monocyte conditioned medium resulted in significant reduction in cell resistance, suggesting the relevance of CCL2 in early immune cell recruitment for subsequent barrier alterations. Further, using Cx3cr1-GFP mice, we found that intraocular injection of CCL2 increased retinal GFP^+^ monocyte/macrophage infiltration. When these mice were made diabetic, increased infiltration of monocytes/macrophages was also present in retinal tissues. Diabetes and CCL2 injection also induced activation of retinal microglia in these animals. Quantification by flow cytometry demonstrated a two-fold increase of CX3CR1^+^/CD11b^+^ (monocyte/macrophage and microglia) cells in retinas of wildtype diabetic animals in comparison to control non-diabetic ones. Using CCL2 knockout (Ccl2^−/−^) mice, we show a significant reduction in retinal vascular leakage and monocyte infiltration following induction of diabetes indicating the importance of this chemokine in alteration of the BRB. Thus, CCL2 may be an important therapeutic target for the treatment of diabetic macular edema.

## Introduction

Diabetic retinopathy is a significant microvascular complication, and is the leading cause of blindness in diabetic individuals in developed countries [1]. An important hallmark of this disease is vascular dysfunction associated with breakdown of the blood-retinal barrier (BRB) resulting in increased retinal vascular permeability and capillary non-perfusion. Previous studies have focused on vascular endothelial growth factor (VEGF) as a mediator of increased retinal vascular permeability, and several clinical trials currently target VEGF for the treatment of diabetic macular edema (DME). Targeting this single molecule appears to have limitations as the improvement is transient, and edema recurs in the majority of patients a few weeks following treatment. In the recent Diabetic Retinopathy Clinical Research (DRCR) study (Protocol I), retinal edema was seen to persist in about 50% of patients with DME even after one year of monthly injections of the anti-VEGF agent, ranibizumab [2]. Based on these results it is possible that other molecules and mechanisms may be operating independently or in conjunction with VEGF in the pathogenesis of this disease.

Accumulating evidence suggests that retinal inflammation plays a major role in the pathogenesis of diabetic retinopathy [3]–[5]. Increased levels of inflammatory mediators may lead to an early, persistent chronic inflammatory condition in the diabetic retina resulting in leukocyte activation, adhesion to the vascular endothelium and extravasation into the retinal tissues [6]–[10]. Hyperglycemic conditions have been shown to up-regulate ICAM-1 (Intercellular Adhesion Molecule-1), a mediator of the adhesion of leukocytes to the endothelium resulting in vascular damage, capillary non-perfusion and changes in vascular permeability [9], [11], [12]. The influx of leukocytes into tissues is stimulated by specific chemokines and their receptors. One of the strongest chemotactic factors for monocytes is CCL2, also known as monocyte chemoattractant protein (MCP-1) [13]. The vitreous levels of CCL2 have been reported to be consistently increased in patients with diabetic retinopathy [14]–17. In addition, the A-2518G CCL2 gene polymorphism, directly associated with increased CCL2 expression, is indicated as a potential risk factor for diabetic retinopathy [18].

In the present study, we hypothesized that increased CCL2 in the diabetic retina alters the BRB indirectly through the recruitment of leukocytes or directly through effects on vascular cells. Results from this study demonstrate that the CCL2 gene is dramatically upregulated in retinas of diabetic animals following the induction of diabetes, and this is coincident with the influx of numerous perivascular monocytes into the retinal tissues. Isolated retinal endothelial cells express increased levels of CCL2 in response to high glucose. Endothelial cells do not increase permeability directly in response to CCL2 under normal conditions. The upregulation of CCL2 appears to play an important role in mediating changes in retinal vascular permeability, presumably through the recruitment of monocytes, as diabetic mice lacking CCL2 are spared these changes compared to wildtype animals. This critical chemokine pathway may thus be an important future therapeutic target for the treatment of DME.

## Research Design and Methods

### Animals and Animal Care

Adult male Sprague-Dawley rats, Cx3cr1-GFP mice (8–10 weeks of age; JAX stock #005582) and CCL2 knockout mice (8–10 weeks of age; Ccl2^−/−^; JAX stock #004434) were used for these studies. Animals were housed in group cages in a pathogen-free environment on a 12 h light/dark cycle and were provided free access to food and water. Animals were euthanized by CO_2_ asphyxiation and eyes enucleated and processed immediately. All animal studies were consistent with and adhered to the ARVO Statement for the Use of Animals in Ophthalmic and Vision Research and were approved by the Institutional Animal Care and Use Committee of the University of New Mexico.

### Animal Model of Diabetes

Diabetes was induced in male rats by a single intraperitoneal injection of streptozotocin (60 mg/kg body weight) in 10 mM citrate buffer (pH 4.5). Mice received five daily consecutive intraperitoneal injections of streptozotocin (50 mg/kg/day). Age-matched non-diabetic control animals received injections of an equal volume of citrate buffer only. Animals with plasma glucose concentrations greater than 250 mg/dL were considered diabetic and were used in the study at 4, 6, or 8 weeks after the induction of diabetes. Blood glucose levels and body weight were monitored regularly. Animals received insulin (0.5 IU) as needed to maintain body weight. The tolerated maximum difference in weight between diabetic and non-diabetic animals was 40%. We also used Cx3cr1-GFP mice crossed with Ccl2^−/−^ mice, and then made these animals diabetic as per the pervious protocol.

### Animal Intraocular Injections

Animals received intraocular injections of PBS (vehicle control) or CCL2 (varying concentrations/eye) under isoflurane anesthesia. The site of injection was at a 3 mm distance from the limbus with a maximum total volume of 10 µL/eye using a 30 gauge needle. Cx3cr1-GFP mice received 50, 20 and 5 ng concentrations of CCL2 in their vitreous cavity, and all concentrations exhibited a similar response. Data for 50 and 20 ng injections are not shown, as human physiological levels range typically from 0.2 to 6 ng, where controls patients are associated with lower levels and higher levels are consistent with proliferative diabetic retinopathy [15]–[17].

### Cell Culture

Human retinal microvascular endothelial cells (HRECs; ACBRI-181) were obtained from Cell Systems (Kirkland, WA). HRECs were grown on fibronectin-coated dishes and cultured in MCDB-131 supplemented with 10% FBS, 10 ng/ml EGF (epidermal growth factor), 1 µg/mL Hydrocortisone, 0.2 mg/mL EndoGro, 0.09 mg/mL Heparin, 100 U/mL penicillin, 100 µg/mL streptomycin and 0.25 µg/mL Fungizone (VEC Technologies, Rensselaer, NY). They were grown to confluence and incubated in MCDB-131 in the presence of either normal glucose (5.5 mM), high glucose (30.5 mM), glucose flux (alternating days of high and normal glucose levels) or the osmotic control mannitol (25.5 mM in normal glucose medium) for 7 days. Cells were harvested for RNA isolation and supernatant medium was collected for ELISA.

Human PBMCs (peripheral blood mononuclear cells) were obtained from Zenbio (Research Triangle Park, NC), and grown on petri dishes in RPMI 1640 culture medium with 10% FBS, 1 mM of L-glutamine, 1 mM of sodium pyruvate, 100 U/mL penicillin and 100 µg/mL streptomycin. To obtain a macrophage-enriched population, cells that were attached to the dish after 3 hours were stimulated with 200 nM PMA (phorbol 12-myristate 13-acetate) for 5 days at 37°C in 5% CO_2_. At that time, 50% by volume of the culture medium was added without replacing the old medium, and cells were allowed to grow for an additional two days. At day seven, the activated macrophage conditioned medium was collected.

### Real Time PCR and PCR Arrays

Total RNA was isolated from retinas or cells (Direct-zol RNA MiniPrep, Zymo Research, Irvine, CA), converted to cDNA (High Capacity cDNA Reverse Transcription; Applied Biosystems, Foster City, CA) and analyzed using the appropriate TaqMan assays (F4/80 and CCL2; Applied Biosystems) by quantitative realtime PCR (qRT-PCR). Relative mRNA levels were determined by the comparative Ct method with normalization to either GAPDH or 18S. The RT^2^ Profiler PCR Arrays were used to examine the expression of pathway focused genes in control and diabetic retinas as per the manufacturer’s instructions (n = 3 animals in each group) (PARN 022 and PARN 024; SA Biosciences, Frederick, MD).

### ELISA

For ELISA, HREC culture supernatants were used to measure CCL2 secreted protein levels (R&D Systems, Minneapolis, MN) and conducted as described by the manufacturer. Samples were diluted accordingly to ensure they were within the dynamic range of the assay, and normalized to total cell number, as estimated by crystal violet staining.

### Confocal Microscopy

Eyes were collected from control non-diabetic Cx3cr1-GFP mice, Cx3cr1-GFP mice with 4 weeks of diabetes (n = 3 animals in each group) and from non-diabetic Cx3cr1-GFP mice 16 hours after an intraocular injection of purified CCL2 (5 ng), (one eye CCL2 injection, and the other, PBS). They were fixed for 2 hours in 2% paraformaldehyde in PBS and retinas removed and incubated for 20 minutes in 70% ethanol/PBS followed by 20 minutes in 1% Triton X-100/PBS. Retinas were then incubated overnight in PBS containing 5 µg/mL AlexaFluor 568-conjugated isolectin GS-IB4 (from *Griffonia simplicifolia*, Molecular Probes, Eugene, OR) to label the vessels and subsequently mounted and examined using a Leica TCS SP5 Spectral confocal microscope (Buffalo Grove, IL) utilizing the LAS AF software (Leica). Images were taken from all areas of the retina, including proximal and distal of the optic nerve. A minimum of 10 photographs were taken from randomized sections of the retina for each mouse. The images shown here are representative of the results seen in all animals of each experimental group.

### Flow Cytometry

Retinas from mouse eyes (n = 4 samples in each group; each sample was composed of 2 animals) were digested with 0.25 mg/mL collagenase D (Roche, Indianapolis, IN) for 30 minutes at 37°C and 100 g/mL DNase I in enzyme free Cell Dissociation Buffer (Life technologies, USA). The tissue digest was then filtered through 70 µm cell strainer, and washed with HBSS buffer with 0.5% BSA for 5 minutes at 400 g at 4°C. The supernatant was carefully removed and the digested tissue pellet was resuspended in FACS buffer to form a single cell suspension. Cell viability was determined by Trypan blue staining method. Viable single cell suspension were then surface stained using fluorescent conjugated monoclonal antibodies CX3CR1-APC and CD11b-PE (eBioscience, San Diego, CA) for 30 minutes at 4°C. Negative controls and single fluorochrome controls were also done for accurate compensation. Flow cytometry data acquisition was done using LSR II (BD Biosciences, San Jose, CA) and analyzed with FlowJo software (Ashland, OR).

### Electrical Cell-Substrate Impedance Sensing (ECIS)

Monolayer permeability was determined using the electrical cell substrate impedance sensing (ECIS) system from Applied Biophysics (Troy, NY). HRECs (1×10^5^) were plated into fibronectin-coated multiwell chambers (8W10E+) and grown for 16 hours until maximum resistance was attained (∼1200 Ω). Purified VEGF (50 ng/ml) or CCL2 (100–500 ng/ml) were added to the wells. Conditioned medium from activated human macrophages (PBMCs) after 7 days of culture, as described above, was added to endothelial cells in a separate experiment. Changes in resistance were monitored for up to 24 hours. Resistance values for multiple wells were normalized to the identical starting resistance value at time 0 and data from 3 wells were averaged and presented as normalized resistance versus time.

### Quantitative Assessment of BRB Permeability

Vascular permeability in the retina was measured using a modification of the Evans blue dye procedure as previously described [19]. Briefly, control or diabetic wildtype and Ccl2^−/−^ mice (n = 3 in each group) were injected via the femoral vein with 45 mg/kg of FITC-labeled albumin, under isofluorane anesthetic (1−3%). The fluorescence of retinal extracts and a 1∶1000 dilution of plasma were measured and the BRB permeability was calculated and expressed as µl/mg·hr/eye.

### Statistical Methods

For all quantitative experiments, statistical analyses of data comparing two separate groups were performed with an unpaired *t*-test, whereas multiple comparisons were done using a one-way ANOVA (Prism4 software; GraphPad). Differences indicated by ANOVA were compared by the Newman-Keuls test. p<0.05 was considered significant.

## Results

### Chemokine Expression Significantly Increases in Diabetic Rat Retinas

**Figure 1:**
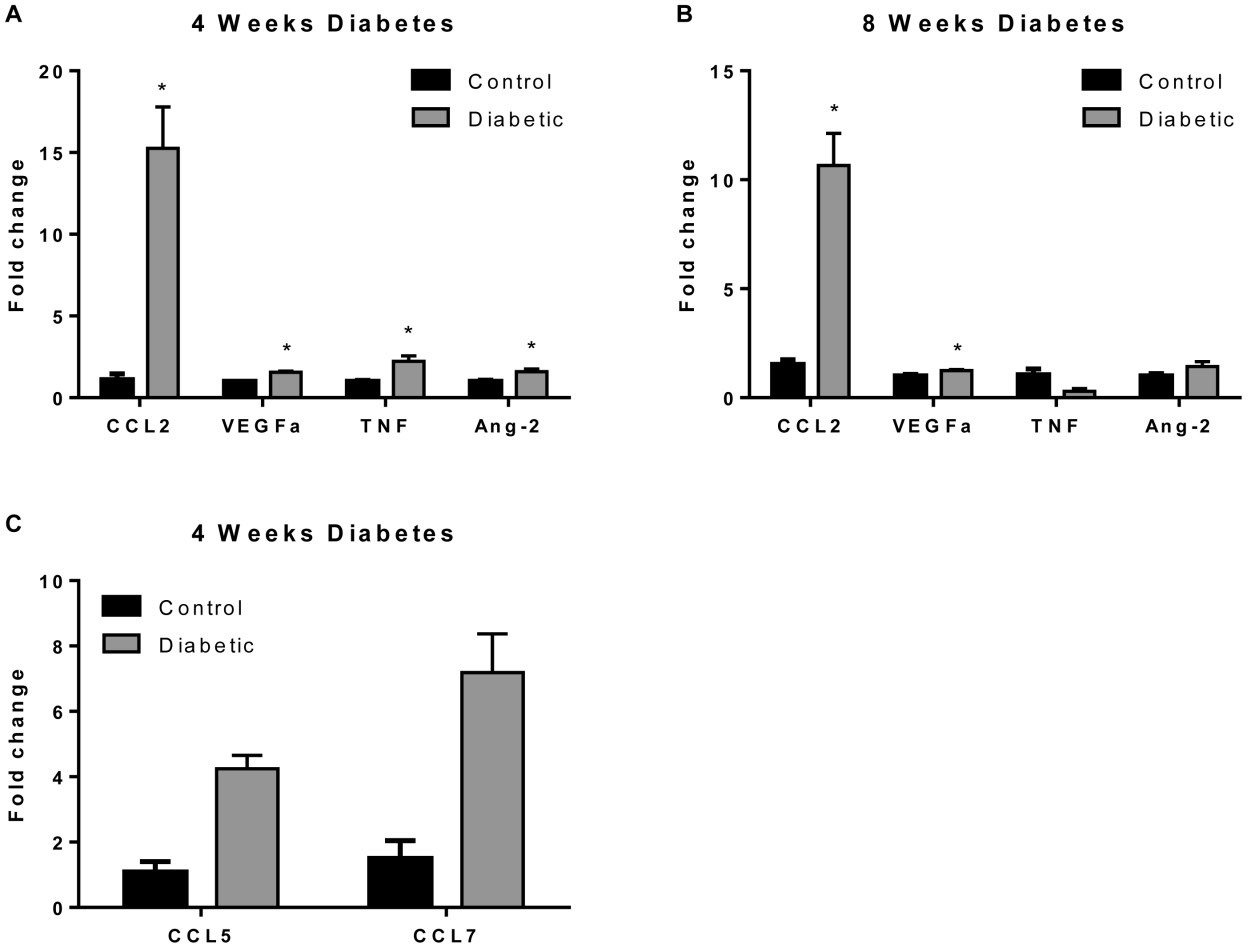
Growth factors and chemokines are upregulated in diabetic rats. PCR gene arrays of 4 week (**A and C**) and 8 week (**B**) diabetic rats demonstrate significant upregulation of a subset of angiogenesis and chemokine genes in comparison to non-diabetic animals. CCL2 is the most remarkably increased in diabetic animals. * Significantly greater than control non-diabetic animals (p<0.05). CCL2: Chemokine Ligand 2; VEGFα: Vascular Endothelial Growth Factor alpha; TNF: Tumor Necrosis Factor; Ang2: Angiopoietin-2; CCL5: Chemokine Ligand 5; CCL7: Chemokine Ligand 7.

Using PCR-based arrays we examined the gene profile of rat retinas with 4 and 8 weeks of diabetes compared to age-matched non-diabetic controls (Fig. 1A). Our analysis focused on growth factors and cytokines with known functions in regulating vascular permeability (VEGFα, angiopoietin-2 (Ang-2), CCL2 and tumor necrosis factor α (TNFα)). After 4 weeks of diabetes the expression of all of these genes was significantly increased with remarkably high levels of CCL2 (15-fold increase; Fig. 1B). Levels of CCL2 and VEGF mRNA, although lower than at 4 weeks, remained significantly elevated after 8 weeks compared to control non-diabetic animals. Further analysis focusing specifically on chemokines and their receptors also revealed a significant increase in the expression of CCL5 and CCL7 in rat retinas with 4 weeks of diabetes (Fig. 1C).

### Retinas of Diabetic Animals Demonstrate Increased Monocyte Trafficking

**Figure 2:**
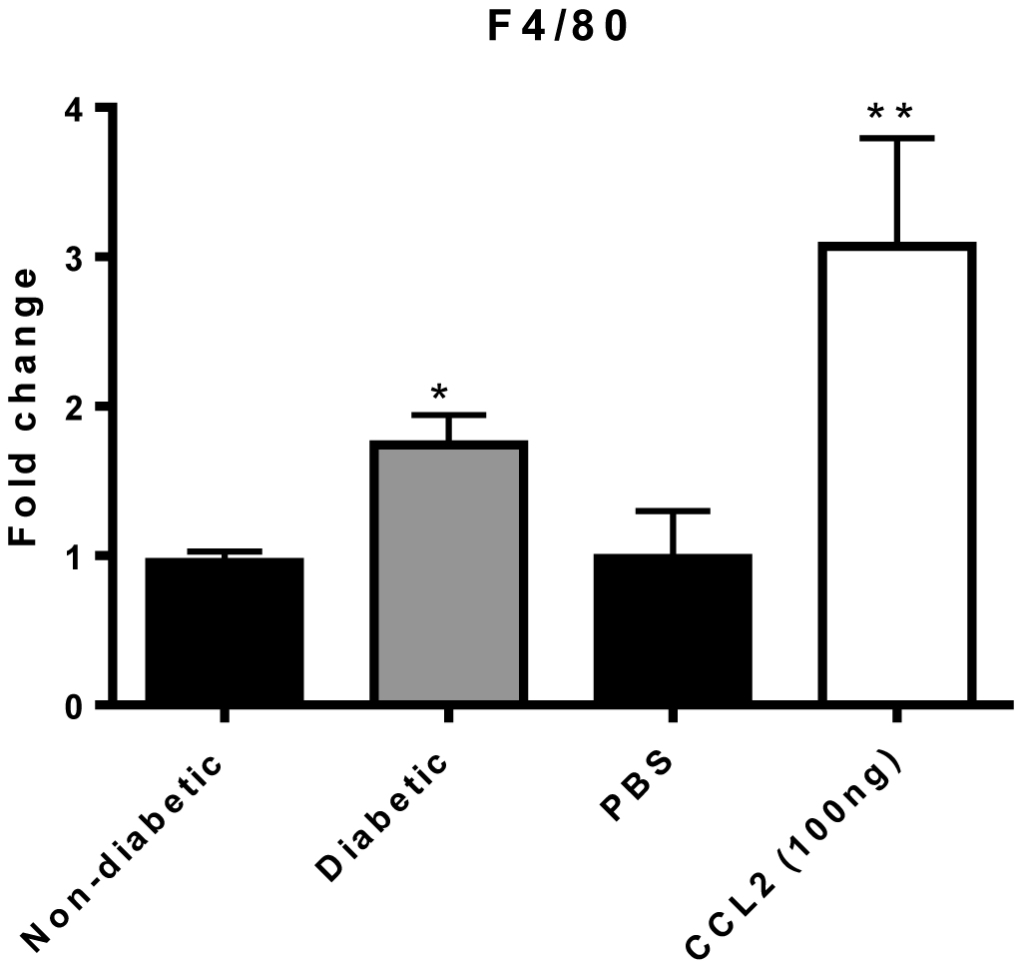
Diabetes or intraocular injection of CCL2 increases F4/80 expression in rat retinas. Levels of the monocyte/macrophage associated marker F4/80 measured by qRT-PCR in 4 week non-diabetic or diabetic rat retinas and rats receiving an intraocular injection of either PBS or CCL2 (n = 4 animals in each group). * Significantly greater than control non-diabetic (p = 0.01). ** Significantly greater than PBS injected (p = 0.035).

Since CCL2 serves as a primary attractant of monocytes to sites of inflammation in tissues, we wanted to determine whether there is infiltration of these cells into the retina in diabetes. To do so, we quantified the monocyte/macrophage specific antigen, F4/80, in retinal tissues of diabetic rats. After 4 weeks of diabetes, retinas demonstrated a nearly 2-fold increase in F4/80 mRNA compared to non-diabetic animals (Fig. 2). In addition, intraocular injection of the chemokine CCL2 into non-diabetic rats resulted in increased retinal expression of F4/80, indicating the ability of this chemokine to attract monocytes/macrophages into this tissue (Fig. 2).

**Figure 3:**
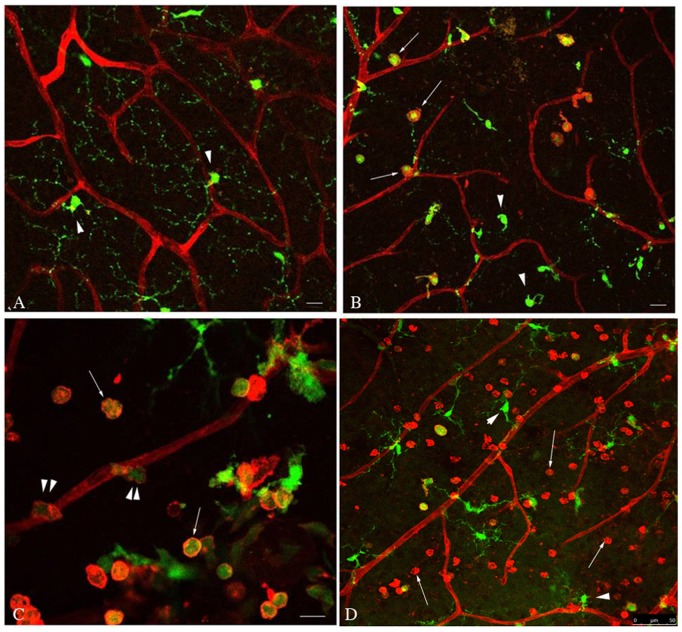
Diabetes or intraocular injection of CCL2 increases monocyte/macrophage infiltration and activates retinal microglia. Representative confocal images of retinal whole mounts from Cx3cr1-GFP mice. Retinas were counterstained with TRITC-labeled GS-IB4 isolectin (red) for vessel and activated monocyte/macrophage identification. (**A**) Normal non-diabetic mice showed isolectin labeled vessels and GFP^+^ retinal microglia (green) (arrowheads) uniformly distributed with ramified processes. (**B**) Retina from a 4 week diabetic animal demonstrates several GFP^+^/isolectin^+^ round cells closely associated with the external surface of the vessels (arrows). In addition, GFP^+^ microglial cells exhibit altered morphology typical of an activated state (arrowheads). (**C**) High magnification image of the retina from a diabetic animal demonstrates co-localization of GFP and isolectin to several of the extravascular monocytes/macrophages within the retinal tissue (arrows). As the isolectin stains activated monocytes in addition to blood vessels, some of the round cells (monocytes/macrophages) also stain red. Further, several GFP^+^ monocytes/macrophages presumably in the process of diapedesis are seen (double arrowheads). (**D**) Mouse retina 16 hours after intraocular injection of purified CCL2 (5 ng). Numerous GFP^+^/isolectin^+^ activated monocytes/macrophages (arrows) and microglia (arrowheads) are present within the extravascular space, with an altered morphology.

The measurement of F4/80 as a marker of monocytes/macrophages in whole retinas does not distinguish whether such an influx of these cells is confined to the lumen of the retinal capillaries, as has been described previously in diabetic animals [20], [21], or whether they have migrated out of the vasculature into the retinal tissues. To address this issue, we utilized the Cx3cr1-GFP mouse model to localize GFP^+^ monocytes/macrophages within the retina [22]. The microglial cells of the retina are also GFP^+^ in this mouse strain but are easily distinguished from monocytes/macrophages by their distinct location and morphology. Retinas from normal non-diabetic mice demonstrated an extensive vascular plexus with no GFP^+^ cells in the extravascular space other than microglial cells (Fig. 3A, arrowheads). There was a unique regular distribution of microglia within defined layers of the retina and these cells demonstrated extensive thin ramified processes with little overlap and uniform spacing between adjacent cell bodies (Fig. 3A). In contrast, retinas from diabetic mice revealed numerous GFP^+^ round cells (monocytes/macrophages), without processes, present within the retinal tissues outside of the vasculature (Fig. 3B, arrows). In some regions, GFP^+^ cells were seen within the lumen of the blood vessels. Many of the cells in the extravascular space were also GS-IB4 isolectin^+^, which has previously been shown to be typical of activated monocytes/macrophages [23]. These monocytes/macrophages were most likely derived from the vasculature as evidenced by the presence of cells that appear to be extravasating from the small vessels into the extravascular space (Fig. 3C, double arrowheads). A similar response was seen in normoglycemic animals injected intraocularly with CCL2 (Fig. 3D). However, the number of monocytes/macrophages in the retinal tissue of CCL2 injected mice was dramatically increased in comparison to diabetic animals, presumably due to the significantly higher concentration of this chemokine administered directly into the eye (Fig. 3D). Retinal microglial cells in both diabetic and CCL2-injected animals revealed an altered morphology with less extensive branching and a reduction in the length of their terminal processes (Fig. 3B and 3D, arrowheads). Especially in the diabetic retinas, microglia took on a typical amoeboid morphology (Fig. 3B), characteristic of its activation [24], [25].

### Flow Cytometry Shows Increase in Monocytes/Macrophage Numbers in Diabetic Retinas

**Figure 4:**
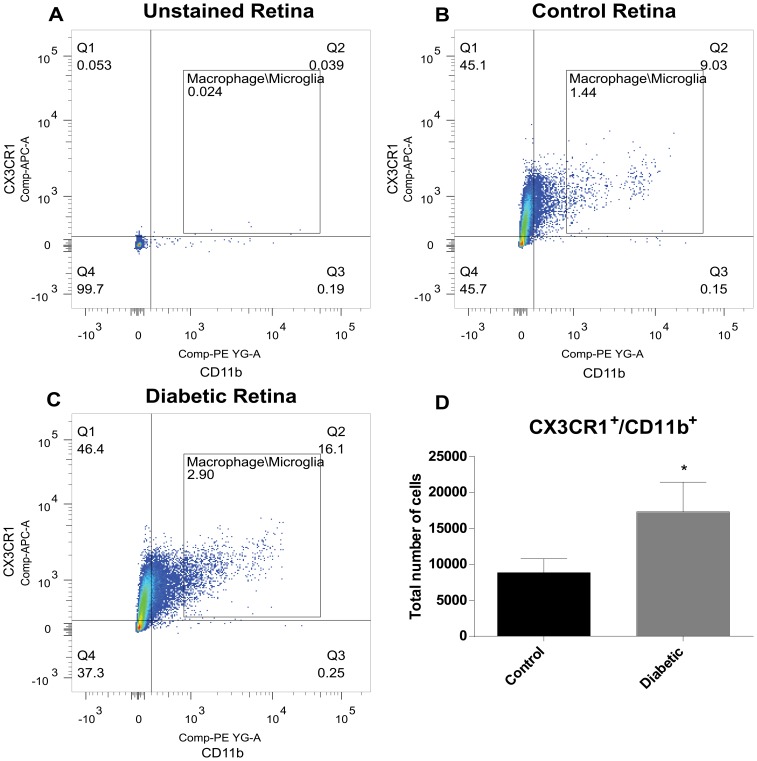
Diabetic mice retinas have significantly higher numbers of CX3CR1^+^/CD11b^+^ macrophage/microglia cells. Representative dot plots of flow cytometry analysis of unstained (**A**), stained control non-diabetic (**B**) and 8 week diabetic wildtype mice (**C**) retinal cell suspensions. Gating was done to include monocyte/macrophage and microglia cells labelled CX3CR1^+^/CD11b^+^. Graphic representation of the total number of CX3CR1^+^/CD11b^+^ cells in diabetic mice demonstrates a two-fold increase in comparison to non-diabetic (**D**). * Significantly greater than control non-diabetic (p<0.05).

To better quantify the amount of infiltrating monocytes/macrophages, we performed Flow cytometry analysis on retinas from 8 week diabetic mice. Since monocytes/macrophages and microglial cells are both derived from the same myeloid cell lineage, it is difficult to find good macrophage-specific markers. In mice, the CD11b antigen is only expressed in these cell types. Unstained cell suspensions from mouse retinas showed minimal staining for CX3CR1 or CD11b (0.024% of all cells, Fig. 4A). Flow cytometry analysis from diabetic retinas (Fig. 4B), compared to control, non-diabetic animals (Fig. 4C) show a significant increase in CX3CR1^+^/CD11b^+^ cells, 2.9% versus 1.44% of total cells, respectively. Total cell counts confirm these observations and are graphically represented in Figure 4D.

### Retinal Endothelial Cells Express and Secrete CCL2 upon High Glucose Stimulation

**Figure 5:**
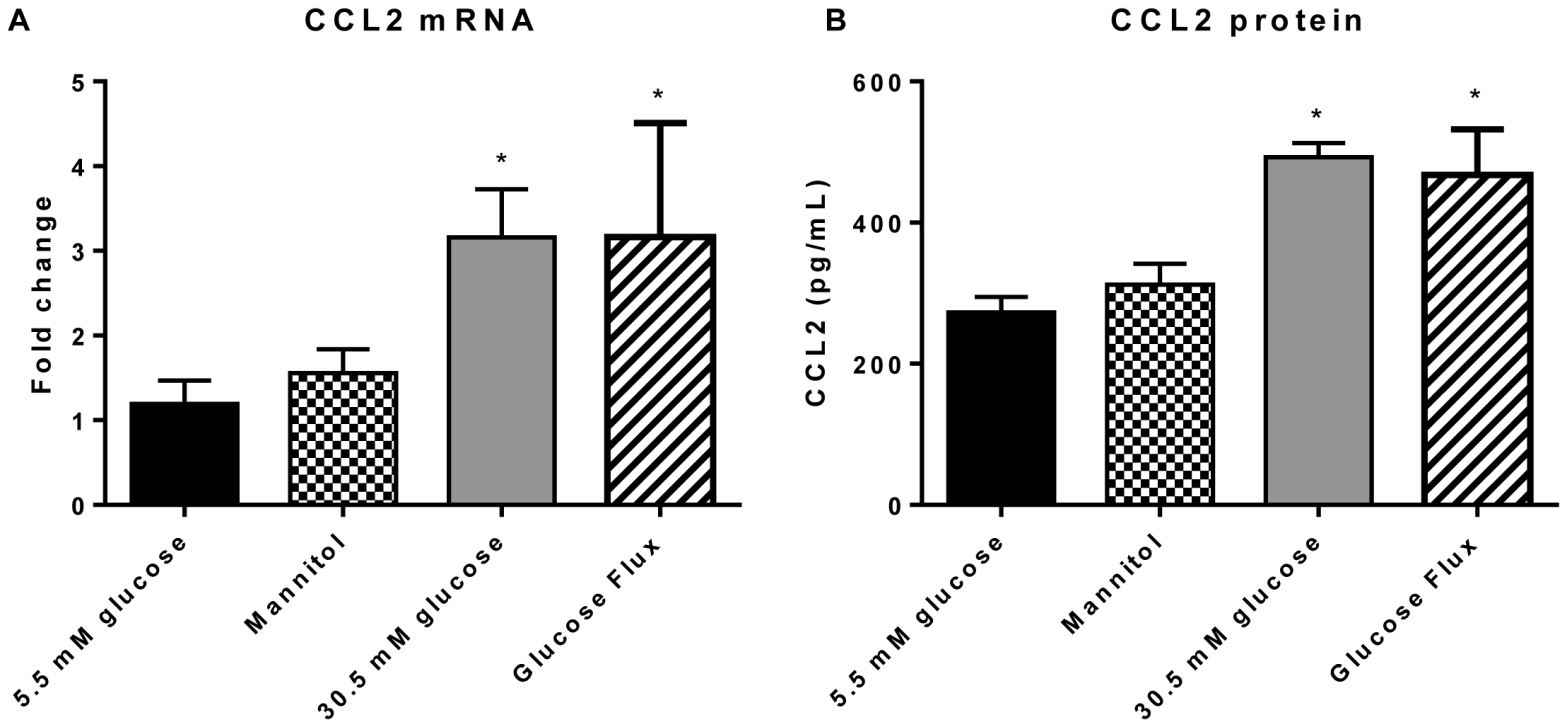
Human retinal endothelial cells (HRECs) express and secrete CCL2 in response to hyperglycemic conditions. HRECs were incubated in normal glucose (5.5 mM), the osmotic control mannitol (25.5 mM), high glucose (30.5 mM) or glucose flux for 7 days. CCL2 gene expression was analyzed by qRT-PCR (**A**), and protein secretion by ELISA (**B**). High glucose and glucose flux significantly upregulated mRNA and protein levels of CCL2 in HRECs. * Significantly greater than low glucose or mannitol-treated cells (p<0.003).

Exposure of isolated retinal endothelial cells to high glucose (30.5 mM) or glucose flux for 7 days resulted in increased expression of CCL2 mRNA (Fig. 5A) or secreted CCL2 protein (Fig. 5B) in these cells compared to normal glucose concentrations (5.5 mM) or the osmotic control mannitol. Thus, our studies indicate that retinal endothelial cells can also contribute to the increased production of the chemokine CCL2 in diabetes, although other cells like Muller cells, pericytes and microglia might contribute to this process.

### CCL2 Does Not Directly Alter Retinal Endothelial Cell Barrier Permeability

**Figure 6:**
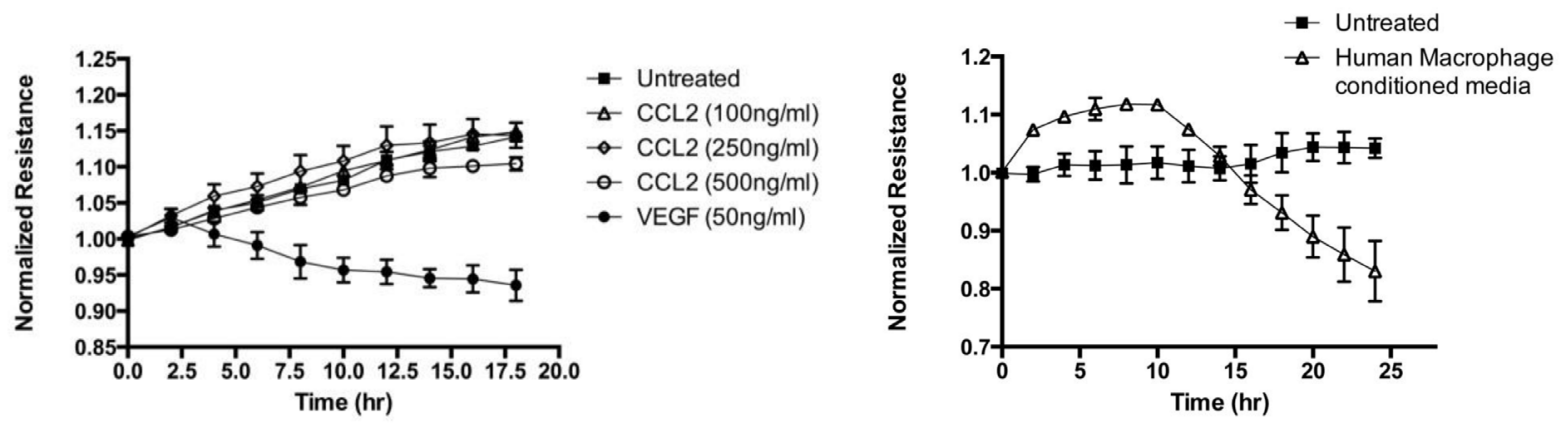
Macrophage conditioned media, not CCL2, alters the integrity of the retinal endothelial barrier. Preformed monolayers of HRECs were incubated with increasing concentrations of CCL2 and VEGF (**A**); or human macrophage (PBMCs) conditioned medium (**B**) and the normalized resistance was determined. No significant change was seen in response to treatment with the different concentrations of CCL2, however, cells incubated with VEGF demonstrated a significant drop in resistance over time. When macrophage conditioned medium was added to endothelial cells, barrier resistance started to decrease significantly after an initial period of 14 hours.

CCL2 may have direct effects on endothelial cells that affect vascular permeability. Previous studies report that ischemia reperfusion injury upregulates CCL2 in brain endothelial cells and astrocytes [26], and treatment of brain endothelial cells with purified CCL2 results in decreased monolayer resistance as measured by TEER (Transendothelial Electrical Resistance) mediated by the redistribution and phosphorylation of tight junction proteins [27]. Using a sensitive measure of monolayer resistance, Electric Cell Substrate Impedence Sensing (ECIS), we were unable to detect a change in response to stimulation with increasing concentrations of recombinant CCL2 (Fig. 6A). These cells, however, responded rapidly and showed significant reduction in resistance in response to VEGF, a well-established inducer of increased endothelial cell permeability (Fig. 6A). Thus, CCL2 does not directly alter the integrity of the retinal endothelial cell barrier. Alternatively, when conditioned medium from activated human macrophages (PBMCs) was added to the endothelial cells, there was significant reduction in resistance after a period of 14 hours (Fig. 6B) suggesting that CCL2 is important for recruitment of inflammatory cells into the retina but that the inflammatory cells themselves may be responsible for the alteration of vascular permeability.

### CCL2 Deficiency Prevents the Increase in Vascular Permeability and Monocyte Trafficking in Retinas of Diabetic Animals

**Figure 7:**
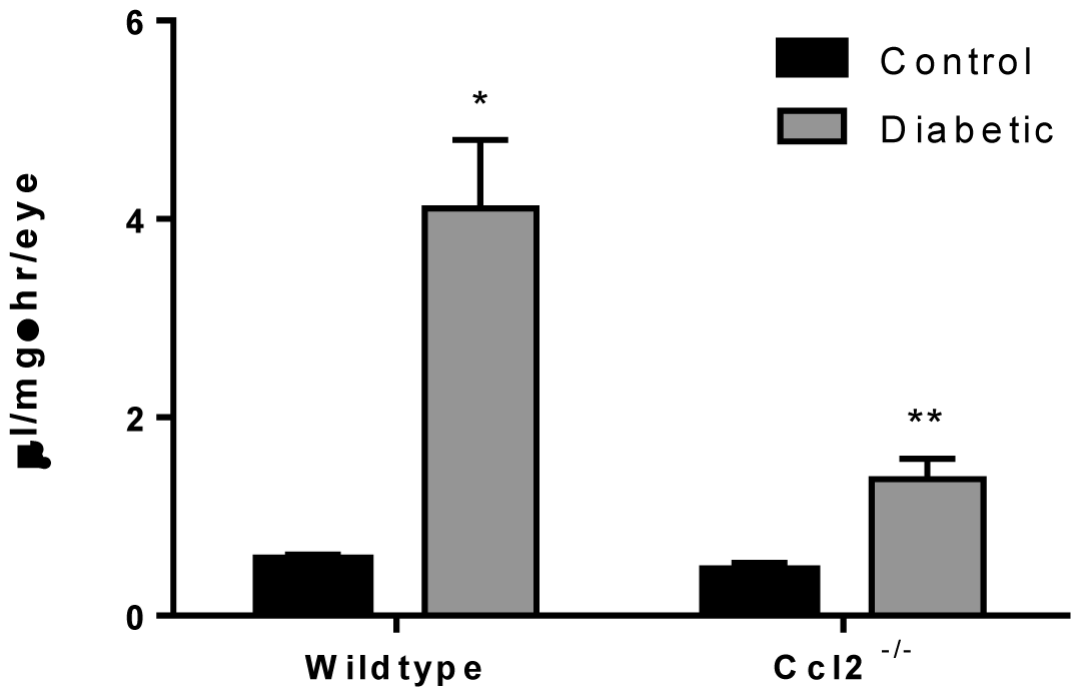
Diabetic Ccl2^−/−^ mice show a reduction in retinal vascular permeability. The modified Evans Blue dye technique was performed in wildtype and Ccl2^−/−^ mice with 6 weeks of diabetes. Wildtype mice demonstrate a nearly 4-fold increase in retinal vascular permeability compared to non-diabetic. The degree of permeability is significantly reduced in Ccl2^−/−^ mice. *Significantly greater than wildtype non-diabetic (p = 0.001). **Significantly less than wildtype diabetic animals and significantly greater than non-diabetic controls (p<0.05).

**Figure 8:**
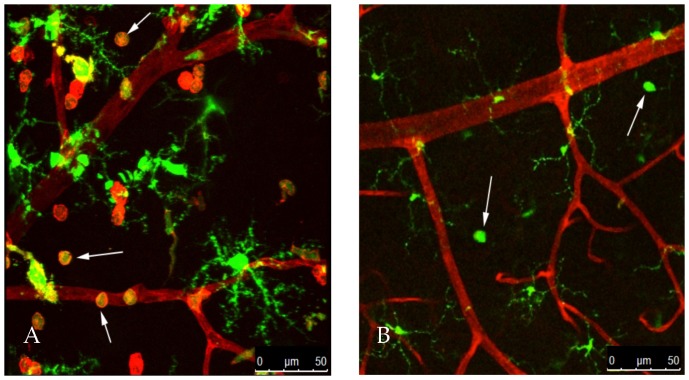
Diabetic retinas of Cx3cr1-GFP/Ccl2^−/−^ mice show significant reduction of activation of microglia and monocyte/macrophage infiltration. Representative confocal images of retinal whole mounts from Cx3cr1-GFP (**A**) and Cx3cr1-GFP/Ccl2^−/−^ mice with 6 weeks of diabetes (**B**) counterstained with TRITC-labeled GS IB4 isolectin for vessel and activated macrophage identification (red). (**A**) Retinas of diabetic Cx3cr1-GFP mice show activated microglia with ameboid morphology (typical of an activated state) and numerous GFP^+^/isolectin^+^ round monocytes/macrophages (arrows). (**B**) Diabetic Cx3cr1-GFP/Ccl2^−/−^ mice show a significant reduction in monocyte/macrophage infiltration. Very few monocytes/macrophages are seen in these retinas (arrows) as opposed to numerous quantities typically seen in diabetic animals (Cx3cr1-GFP). Retinas show GFP^+^ microglia uniformly distributed with ramified processes, similar to inactivated cells.

Retinal permeability was measured in Ccl2^−/−^ mice by a modified Evans Blue dye technique following 6 weeks of diabetes. Compared to wildtype mice, that exhibited a four-fold increase in permeability, mice lacking CCL2 demonstrated only a 1.5-fold increase (Fig. 7). To investigate whether such a decrease in retinal vascular permeability is related to monocyte/macrophage trafficking in the retinas, we examined Cx3cr1-GFP mice crossed with Ccl2^−/−^ mice made diabetic for 6 weeks. In comparison to diabetic Cx3cr1-GFP mice (Fig. 8A), the retinas of the Ccl2^−/−^ animals showed a significant reduction (almost complete absence) of GFP^+^/isolectin^+^ monocytes/macrophages (Fig. 8B). Additionally, microglia in the knockout mice showed an inactivated morphology with long ramifying processes. These results indicate that the chemokine CCL2 plays a significant role in increased retinal vascular permeability seen in diabetes, and based on earlier data, suggests that the presence of monocytes/macrophages in retinal tissues is critical for this response.

## Discussion

Our findings demonstrate that specific chemokines are upregulated in the retinas of diabetic animals, and that the chemokine CCL2, plays an indirect role in mediating the increase in retinal vascular permeability in diabetes. Gene expression array results indicate that although VEGF is upregulated in diabetic retinas along with other angiogenic factors like Ang-2 and TNFα, CCL2 is remarkably upregulated, and this increase is maintained at least up to 8 weeks of diabetes. Our findings in the retinas of diabetic animals are consistent with similarly increased levels of CCL2 seen in the vitreous of patients with diabetic retinopathy [14]–[17].

Further analysis by gene array also showed that among all members of the chemokine family, CCL5 and CCL7 are significantly upregulated. Chemokines have an important role in host defense by regulating infiltration of passing leukocytes [28]. More than 45 chemokines have been identified, binding to glycosoaminoglycans on the surface of endothelial cells, thus rapidly forming a solid-phase chemotactic gradient to attract passing leukocytes. Currently, 19 chemokine receptors have been described, and are expressed on immune cells, endothelial cells and neurons. Each receptor has a repertoire of chemokine ligands responsible for its activation. The majority of previous work focuses on the expression of chemokines and receptors in other inflammatory diseases including atherosclerosis, multiple sclerosis, rheumatoid arthritis, lupus, obesity and age-related macular degeneration [29], [30]. Less is known specifically about chemokines and their receptors in the retinal microvasculature in diabetes. CCL2 has been shown to play a role in retinal neovascularization [31] and retinal detachment [32]. An earlier report describes the immunolocalization of the chemokines, CCL2 and CCL5, in the inner retina of human diabetic donor eyes [33]. Furthermore, the A-2518G CCL2 gene polymorphism, shown to be a potential risk factor for patients with diabetic retinopathy, strengthens the evidence for a role of this chemokine in diabetic macular edema [18].

The importance of leukocyte recruitment and adhesion leukostasis in mediating retinal vascular permeability has been described in previous studies [4], [5]. Elevated levels of neutrophils have been shown in both retinal and choroidal vessels of patients with diabetes [9]. Our study shows both increases in expression of F4/80, a monocyte/macrophage associated marker, increased CX3CR1^+^/CD11b^+^ cells, as well as infiltration of activated monocytes/macrophages into the retinas of diabetic animals, confirming prior work in which activated monocytes and neutrophils were encountered in the retinas of diabetic rats and monkeys [8], [10]. Using chimeric mice, bone marrow derived cells (neutrophils and monocytes) have been highly associated with retinal capillary degeneration in diabetes [34]. Also, CD11b^+^ bone marrow derived monocytes are the major leukocyte subset entrapped in the retinal microcirculation during leukostasis in diabetic mice [35]. Additionally, our study has shown evidence for increased CX3CR1^+^/CD11b^+^ cells and monocyte/macrophage trafficking into retinal tissues of diabetic animals. The presence of activated monocytes/macrophages in the extravascular space is evidence of the ability of leukocytes to breach the BRB by diapedesis and establish an inflammatory reaction within the retinal tissues. On several occasions, we observed monocytes/macrophages adhering to the outer surface of retinal capillaries, most likely in the active process of extravasation.

Activated monocytes, once present in the extravascular space, differentiate into macrophages that secrete cytokines and growth factors including VEGF, Ang-2, TNFα and interleukins. Breakdown of the BRB leading to increased vascular permeability has been attributed to increases in these cytokines and growth factors [7], [36], [37]. In this study we have demonstrated that conditioned medium from human macrophages can alter the resistance of the retinal endothelial cell monolayer. The macrophage conditioned medium contains several cytokines including TNFα, CCL2, CCL5, IL-6, and IL-8 [38]. Further characterization of the factor(s) responsible for this increased permeability in our ECIS study is in progress. The initial delay in response of the macrophage conditioned medium for reduced resistance of the endothelial cells in our system is probably due to different processing times necessary for different signaling pathways for specific cytokines involved in the inflammatory cascade. The presence of monocytes/macrophages can also lead to the production of extracellular proteinases including matrix metalloproteinase 2 and 9 (MMP2 and MMP9), which have been shown to alter the integrity of the BRB [39]. Our findings also indicate that retinal microglia become similarly activated in both CCL2 injected eyes and in diabetic animals. Retinal microglia normally display a continuous, dynamic behavior suggestive of tissue surveillance or intercellular communication functions [7]. A similar morphological change of retinal microglia has also been described after focal laser injury, where they rapidly transition from a mainly symmetrical shape into a more polarized phenotype towards the laser lesion [40]. Morphologic changes of retinal microglia that occur in early diabetes suggest that they may have an inductive role in causing pathological neuronal and vascular changes.

Previous studies have reported that Muller cells and microglia can upregulate the expression of CCL2 in response to light-induced damage and to inflammatory cytokine stimulation [41], [42]. Here, we demonstrate that isolated retinal endothelial cells can be an additional source of CCL2, occurring in response to high glucose conditions. A similar increase in CCL2 expression by Muller cells, microglia and pericytes in response to high glucose has not yet been determined. In diabetes, the increased expression of CCL2 and other chemokines in the retina may be due to upregulation by any or all of these cell types.

Our study shows significantly less retinal vascular leakage and decreased monocyte/macrophage trafficking into diabetic Ccl2^−/−^ retinas, indicating that this chemokine may be essential for the alteration of the BRB. Based on our data we conclude that this effect is mediated primarily by the attraction and influx of monocytes/macrophages into the retina and simultaneous activation of microglia with release of several cytokines that affect the cell-cell tight and adherens junctions of the retinal vascular endothelium, leading to the alteration of the blood-retinal barrier. Although the gap junctions have been traditionally described to facilitate cell-cell communication, a recent study indicates that high glucose-induced downregulation of the gap junction, connexin 43 contributes to the breakdown of tight junctions this indicating a potential mechanism for the altered BRB [43]. However, the CCL2 has limited direct effects on the endothelium itself. Therefore, it is tempting to speculate that pharmacological targeting of CCL2 may be a safe and viable strategy in treatment of DME. The efficacy of steroids in reducing retinal thickness and preventing vision loss in DME, as shown in the DRCR Network Study, indicates that the inflammatory cascade can be an effective target in the treatment of this disease. However, steroids have serious side effects, including cataract formation and glaucoma [2]. Whereas most of the current clinical trials target the molecule VEGFα, targeting CCL2 can address the mechanisms of early leukocyte influx, leukostasis and adhesion to the retinal capillary wall in diabetes, a potentially critical upstream target for the treatment of DME. Therapeutic targeting approaches have been utilized for at least 9 chemokines and their receptors in different diseases including atherosclerosis, multiple sclerosis, diabetic nephropathy and diabetes itself [29]. Accumulating evidence suggests that the chemokine/chemokine receptor axes can be targeted for treating inflammatory diseases like atherosclerosis, multiple sclerosis, rheumatoid arthritis and age-related macular degeneration [28]–[30], [44]. CCL2 targeting alone or in combination with the current anti-VEGFα therapy or laser, may be a more specific inhibition strategy for reducing vision loss in DME.
